# Impact of endodontic access cavity preparation on the fracture resistance of CAD-CAM crowns

**DOI:** 10.1186/s12903-025-05934-2

**Published:** 2025-07-11

**Authors:** Abdelazim Gamal, Mohamed F. Aldamaty, Hussein R. Mohamed, Hesham I. Othman

**Affiliations:** Department of Fixed Prosthodontics, Faculty of Dental Medicine, Al-Azhar University 11651, Cairo, Egypt

**Keywords:** Ceramic repair, Endodontic access cavity, Failure mode, Fracture resistance, Lithium disilicate, Polymer-infiltrated ceramic, Thermo-mechanical loading

## Abstract

**Purpose:**

The present research aimed to assess the impact of endodontic access cavity preparation on the fracture resistance of CAD-CAM crowns.

**Materials and methods:**

A total of 40 extracted human upper first premolars were utilized in present research. All premolars were affixed in epoxy resin blocks, prepared by utilizing a CNC milling machine to receive full coverage ceramic crowns, and evenly split into two primary groups based on the type of ceramic; Group LD: teeth restored with lithium-disilicate (LD) crowns, and Group PIC: teeth restored with polymer-infiltrated ceramic (PIC) crowns. Every group was subdivided into 2 subgroups (*n*=10); Subgroup LDI: Intact LD crowns, Subgroup LDR: Repaired LD crowns, Subgroup PICI: Intact vita PIC crowns, and Subgroup PICR: Repaired PIC crowns. Crowns were cemented using Calibra Universal resin cement. The repaired subgroups received a standardized access cavity at the center of the occlusal surface and then repaired with direct composite resin. All samples were exposed to thermo-mechanical loading in a chewing simulator for 118,000 cycles, loaded until failure, and then statistically analyzed.

**Results:**

For intact control subgroups, the greatest mean scores were showed in PICI (1308.71±244.15 N) compared to LDI (1154.38±133.83 N), and the variation was not statistically significant (*P*=0.097). For repaired subgroups, the highest mean values were recorded for PICR (727.84±240.52 N) compared to LDR (707.03±298.28 N), and the variation was not statistically significant (*P*=0.866).

**Conclusions:**

Both LD and PIC crowns perform the same after exposure to an endodontic access cavity, suggesting their repairability and useability.

## Introduction

Whenever trauma and/or carious lesion cause a significant loss of coronal dental tissue, a full-coverage crown restoration might be necessary. Esthetic ceramic restorations are in increasing popularity as a result of the latest advances in the field of CAD-CAM technologies. Full-coverage crown restorations are not considered conservative operations; they may need the removal of approximately 24 and 70 percent of the tooth's natural structure [1]. Therefore, the vital teeth restored with crowns may eventually need root canal treatment as a result of exposing pulp tissue to inflammatory mechanisms that include heat, mechanical stress, and chemical insults [2, 3].

According to Cheung et al. [3], 38% of teeth prepared for fixed dental prostheses (FDPs) and up to 6% of single crowns eventually needed root canal therapy after definite cementation of restoration, while other studies estimated that the incidence may range from 0.7–21 % [4–9].

The dentist's clinical judgment will determine whether to remove the entire restoration or create access through the existing crown if root canal therapy is deemed necessary [10]; nevertheless, it is essential to thoroughly assess the risks associated with performing the root canal procedure through the crown. Firstly, because the crown obscures the coronal tooth anatomy, finding the pulp chamber might be difficult [11]. Secondly, to guarantee the long-term effectiveness of restoration and root canal therapy, the sealed access cavity is essential for preventing microleakage [12]. Lastly, the accessible crown's ability to withstand fractures may be jeopardized [11, 13].

Prior investigations assessing how simulated endodontic access cavity preparation affects ceramic crown fracture resistance has identified a number of parameters including the selection of luting cement [13], how the access cavity is repaired and filled, and the grit size of the rotating tool utilized for access preparation [14], and as the variable that is most usually studied,"the kind of ceramic material employed” [11, 13–16].

Prior studies were conducted on a variety of ceramic materials with root canal access cavity simulations [10, 11, 16, 17]. When Wood et al. [11] examined two different polycrystalline ceramic materials, they found that zirconia crowns'fracture resistance significantly decreased following the root canal access simulation, not the alumina crowns. But the glass-matrix ceramic substances produced outcomes that were disputed. According to Bompolaki et al. [16], there was a noticeable reduction in fracture resistance when pressed lithium disilicate crowns were prepared for root canal access. On the contrary, there is no difference amongst intact and endodontically accessible lithium disilicate crowns, according to Gerogianni et al. [10].

Since the introduction of CAD-CAM blocks of polymer infiltrated ceramics (PICs), clinicians favor the material due to their rapid manufacturing and lack of need for a crystallization phase following the milling procedure [18]. Furthermore, due to their low hardness values, the PICs stand out for their properties of minimum peripheral chipping and avoidance of abrasion of the antagonist natural teeth [18–20]. Many research assessing polycrystalline and glass-matrix ceramic crowns have already been carried out. Nevertheless, there aren't many published studies examining how endodontic access simulation affects PIC restorations. Consequently, the null hypothesis of the present research was that “the endodontic access cavity preparation through ceramic crown wouldn't have an impact on the fracture resistance of ceramic restorations using polymer-infiltrated polymer and lithium disilicate”.

## Materials and methods

Based on a prior investigation by Bompolaki et al. [16] and by utilizing the G power statistical power analysis application (version 3.1.9.4) to calculate the appropriate sample size. For a two-sided hypothesis test, a total sample size of 40 would be adequate to identify a high effect size (d)= 1.05, having an actual power (1-β error) of 0.9 (90%) and a level of significance (α error) of 0.05 (5%). The samples were divided into 20 in each group according to ceramic material (IPS e.max CAD and Vita Enamic), then subdivided into two subgroups (*n*= 10), intact (control) and repaired crowns.

A total of forty recently extracted human maxillary 1^st^ premolars were gathered from orthodontic and individuals who have periodontal disease. For this investigation, teeth with average similarities in terms of dimensions, form, and root anatomy were chosen. The average tooth dimensions were (8.5 ± 0.5 mm) in occluso-cervical length, (8.0 ± 0.5 mm) in bucco-palatal diameter, and (5.0 ± 0.5 mm) in mesiodistal diameter.

After being gathered, the premolars were cleaned from any debris and disinfected. Using a magnifying lens, teeth were inspected under proper lighting to ensure that the teeth had complete root and crown morphology, sound, and caries-free and had no cracks, hypoplastic defects, or restorations. Then each tooth was mounted in an epoxy resin block. The roots of the teeth were centrally mounted and inserted in self-curing epoxy resin (Exakto-Form, Bredent, Germany) and held 2.0 mm away from the cemento-enamel junction utilizing a parallometer (Parallometer, Bego, Bremer, Germany).

**Fig. 1:**
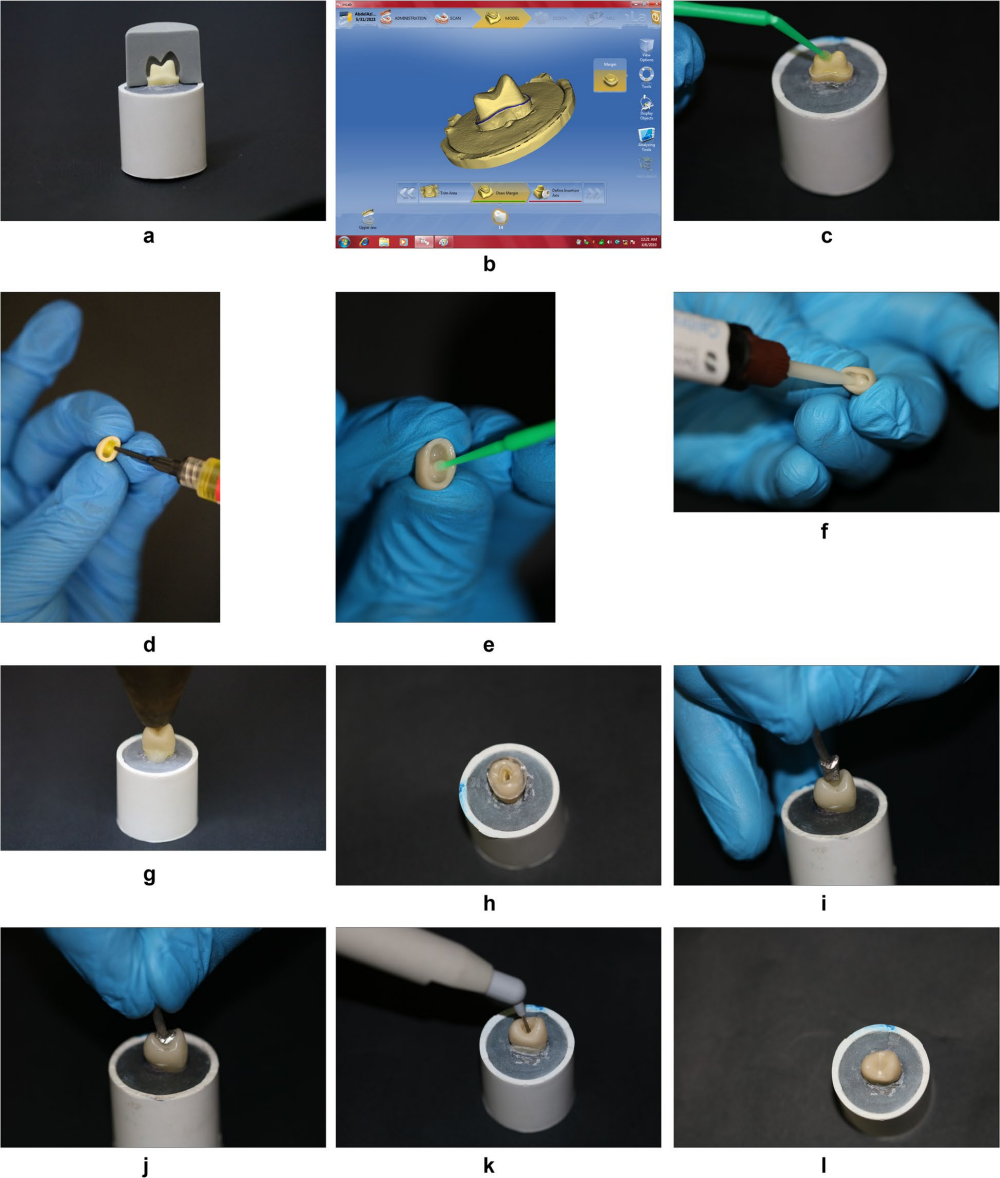
Practical steps of the study: **a** Prepared abutment, **b** Scan and design by CEREC inLab system, **c** Abutment conditioning, **d** Etching of ceramic crown by 9.5% HF, **e** Application of silane coupling agent after etching, **f** Application of resin cement, **g** Cementation of ceramic crown to the conditioned abutment using static load, **h** Perforated plastic template adapted on the occlusal surface of the crown as a guide for oval-shape access cavity, **i** Metal inlay fabricated to act as a guide for 5 mm depth, **j** Metal inlay in situ, **k** Repairing access cavity with bulk fill composite resin, **l** Repaired crown after polymerization and finishing of composite resin

A particular computer numerical control (CNC) (Premium 4820, imes-icore, Eiterfeld, Germany) was used for standardization during tooth preparation to receive full coverage ceramic crowns (Fig. 1). All teeth were prepared with 2.0 mm occlusal reduction, 1.5 mm axial reduction, 12° occlusal convergence angle, and 1.0 mm circumferential chamfer finish line [21]. The prepared premolars were split equally into 2 groups randomly (20 each) based on the ceramic type; Group LD: Premolars restored using lithium-disilicate crowns (IPS e.max CAD, Ivoclar Vivadent, Schaan, Liechtenstein), and Group PIC: Premolars restored using polymer-infiltrated ceramic crowns (Vita Enamic, Vita Zahnfabrik, Germany). Every group was further subdivided into 2 subgroups (*n*= 10); Subgroup LDI: Intact lithium disilicate crowns (control), Subgroup LDR: Repaired lithium disilicate crowns, Subgroup PICI: Intact polymer-infiltrated ceramic crowns (control), and Subgroup PICR: Repaired polymer-infiltrated ceramic crowns.

The prepared teeth were sprayed with Cerec Optispray (Sirona Dental Systems GmbH, D- 64625 Bensheim, Germany), scanned with inEos blue scanner (Sirona Dental Systems GmbH, D- 64625 Bensheim, Germany), crown restoration designed by Cerec inLab 3D software version 4.2 (Sirona Dental Systems GmbH, D- 64625 Bensheim, Germany), and finally restoration milled by MC XL milling unit (Sirona Dental Systems GmbH, D- 64625 Bensheim, Germany) from IPS e.max CAD blocks for LD group and Vita Enamic blocks for PIC group. Crystallization was performed in the Programat P310 furnace (Ivoclar Vivadent AG, Schaan/Liechtenstein) for IPS e.max CAD crowns in compliance with the supplier's guidelines, while Vita Enamic crowns were just polished utilizing the Vita Enamic clinical 2-step polishing Kit (Vita Enamic Polishing Set clinical VITA, Vita Zhanfabrik. Germany) in compliance with the supplier's guidelines. After that, each milled crown was examined to make sure it fit well on their corresponding abutment.

All crowns were ultrasonically cleaned for 4 minutes [22] to guarantee a very clean surface for cementation. The fitting surface of LD crowns was treated for 20 sec with porcelain etch (hydrofluoric acid 9.5%, BISCO, USA) and to PIC crowns for 30 sec. [23] after that washed with water and dried using dry, oil-free air till the crown's internal surface showed a frosted white look. Then, the porcelain silane coupling agent (Porcelain Primer, BISCO, USA) was applied with a brush to the etched ceramic surface and left for 60 sec. then dried well. A single-bottle adhesive (prime & bond universal, Dentsply, Sirona) was used for conditioning the internal surfaces of all crowns and the external surfaces of abutment teeth. Dual-cure self-adhesive resin cement (Calibra Universal, Dentsply, Sirona) was then utilized to finish the cementation. The applied static load was standardized by 4 kg using a specially designed apparatus throughout cementation [24]. Light curing for 2 seconds per surface was performed in compliance with the supplier's guidelines. After that, all the extra cement was eliminated. Cement was fully polymerized by the use of a light-curing device (700 Mw/Cm^2^. 3M Elipar Deep Cure-L LED Curing Light USA) from five directions and for 100 seconds (20 seconds per surface) [21].

Ten specimens from each group received a standardized endodontic oval-shaped access cavity (3.5 × 2.5 mm) with a 5.0 mm cavity depth. To standardize the oval shape of the access cavity on all repaired crowns, a polyvinyl siloxane sheet was pressed on the initial sample under vacuum and heating for fabrication of a plastic template and marked on the occlusal surface then perforated to act as a guide during access cavity preparation [21]. On the initial sample, a conservative oval-formed access was indicated, and processing was carried out in accordance with these marks. Following the preparation was completed on the initial sample, a metal-inlay was fabricated for the access cavity using the lost wax technique. This metal-inlay serves as a guide to standardize the 5.0 mm cavity depth for all repaired specimens.

The same clinician performed all endodontic access preparations using a high-speed handpiece operated at 200,000 rpm under abundant water irrigation. Two rotary instruments were used; a round diamond bur (ZR6801.314.014, Komet, Germany) for initiating the endodontic access and perforating the restoration, and a cylindrical diamond bur (ZR6856.314.025, Komet, Germany) for finishing the preparation. Every cavity preparation procedure employed a brand-new diamond rotary tool [16].

Crown samples were repaired using the porcelain repair method including hydrofluoric acid gel 9.5%, silane coupling agent, Prime & Bond Universal, and direct nanocomposite resin (Dentsply SDR^®^ Plus Bulk Fill Flowable Composite). Surface treatment was applied for both ceramics in compliance with the supplier's guidelines, Prime & Bond was applied on the access cavity and light-cured, then nanocomposite restoration was inserted in two layers (2 mm each), and every layer was light-cured for 20 sec. [21]. The neighboring crown material was used to level the occlusal section of the repair, and a rubber wheel composite polishing with water coolant was used to gently smooth the contact. For one day, all samples (Intact and Repaired) were kept in an incubator with 100% humidity and 37 °C [21].

To represent one year of clinical service, all samples (Intact and Repaired) were subjected to thermo-mechanical loading in a chewing simulator (Robota, Model ACH- 09075DC-T, ADTECH Technology Co., LTD., Germany) for 118,000 cycles with the application of 98 N of a chewing force, 2.4 Nm torque, and 1.6 Hz cycle frequency. Cold/hot bath temperature: 5/55 °C with 60 sec. dwell time [25].

All specimens (Intact and Repaired) were put separately on a universal testing device (Model 3345; Instron Industrial Products, Norwood, MA, USA). Only one static compressive force was put on for every crown throughout the long axis until a fracture happened. A steel rod with a rounded tip (4.0 mm in diameter) that was located on the occlusal surface and pointed towards every crown's central groove was used as the force applicator. Utilizing computer software, the force necessary to cause failure was measured for every sample and reported in Newtons (N).

The failure mode was inspected utilizing a stereomicroscope and scanning electron microscope (SEM). The fracture mode of the crowns was categorized based on the Burke’s classification [26]; Class I: a little crack or fracture in the crown Class II denotes a loss of less than half of the crown; Class III, a fracture across the center and half of the crown broken or dislocated; Class IV, a loss of greater than half of the crown; and Class V, an extensive fracture of the tooth and/or crown.

The statistical analysis and data management were conducted using the Statistical Package for Social Sciences (SPSS, version 20, IBM Co. USA). Utilizing mean, standard deviation, median, and range, numerical information was presented. Kolmogorov-Smirnov and Shapiro-Wilk tests were used to examine the normality of the data.

## Results

Based on the normal distribution of the data, fracture resistance values were compared between groups utilizing an independent t-test and ANOVA test. Paired t-test was employed for intra (within) group comparisons. All *P*-values were two-sided. *P*-values ≤ 0.05 were regarded as significant.

PICI has the highest mean value of fracture resistance (1308.71 ± 244.15 N), followed by LDI (1154.38 ± 133.83 N), then PICR (727.8 ± 240.52 N), whereas LDR had the lowest mean value (707.03 ± 298.28 N). ANOVA test displayed that the variation between subgroups was statically significant (*P*< 0.001). Post hoc test showed that there was no significant variation between the control subgroups (PICI and LDI). Moreover, there was no significant variation between the repaired subgroups (PICR and LDR) (Table 1).

**Table 1:** Descriptive statistics and comparison of fracture resistance between different subgroups

**Groups**	**Mean**	**Std. Dev**	**95% Confidence Interval for Mean**		**Min**	**Max**	**F**	***P* value**
			**Lower Bound**	**Upper Bound**				
**LDI**	1154.38 ^a^	133.83	1058.65	1250.12	923.64	1343.21	16.43	< 0.001*
**PICI**	1308.71 ^a^	244.15	1134.05	1483.36	976.21	1623.82		
**LDR**	707.03 ^b^	298.28	493.66	920.41	409.16	1178.41		
**PICR**	727.84 ^b^	240.52	555.78	899.90	429.18	1027.97		

Significance level *P*≤ 0.05, *=significant

Post hoc test: means sharing the same superscript letter are not significantly different

*LDI* Lithium disilicate-Intact, *PICI* Polymer Infiltrated Ceramic-Intact

*LDR* Lithium disilicate-Repaired, *PICR* Polymer Infiltrated Ceramic-repaired

### Failure mode of IPS e.max and Vita Enamic ceramic crowns: (Figs. 2, 3, and 4)

**Fig. 2:**
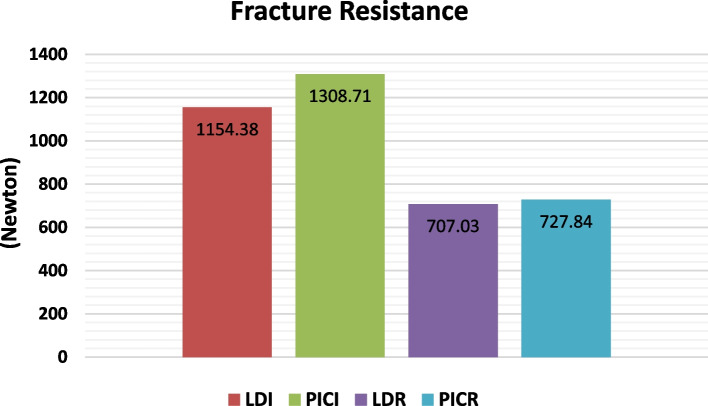
Failure mode distribution (%) for control and repaired groups

**Fig. 3:**
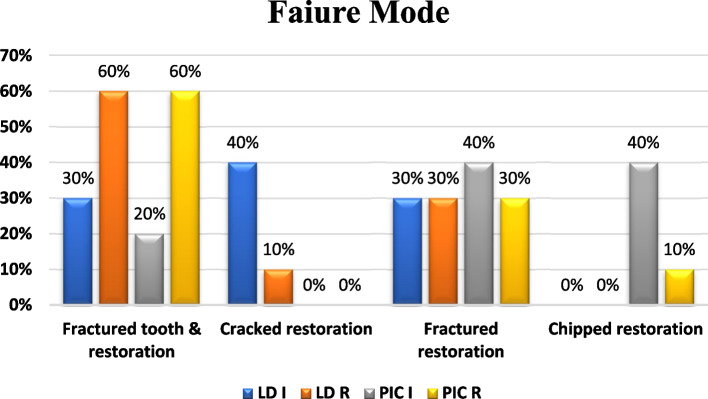
Scanning Electron Microscope images (SEM) showing different failure modes. **a** Cracked IPS e.max crown (Control group), **b** Fractured restoration and tooth in IPS e.max (Repaired group), **c** Fractured restoration in Vita Enamic (Control group), **d** Fractured restoration and tooth in Vita Enamic (Repaired group

**Fig. 4:**
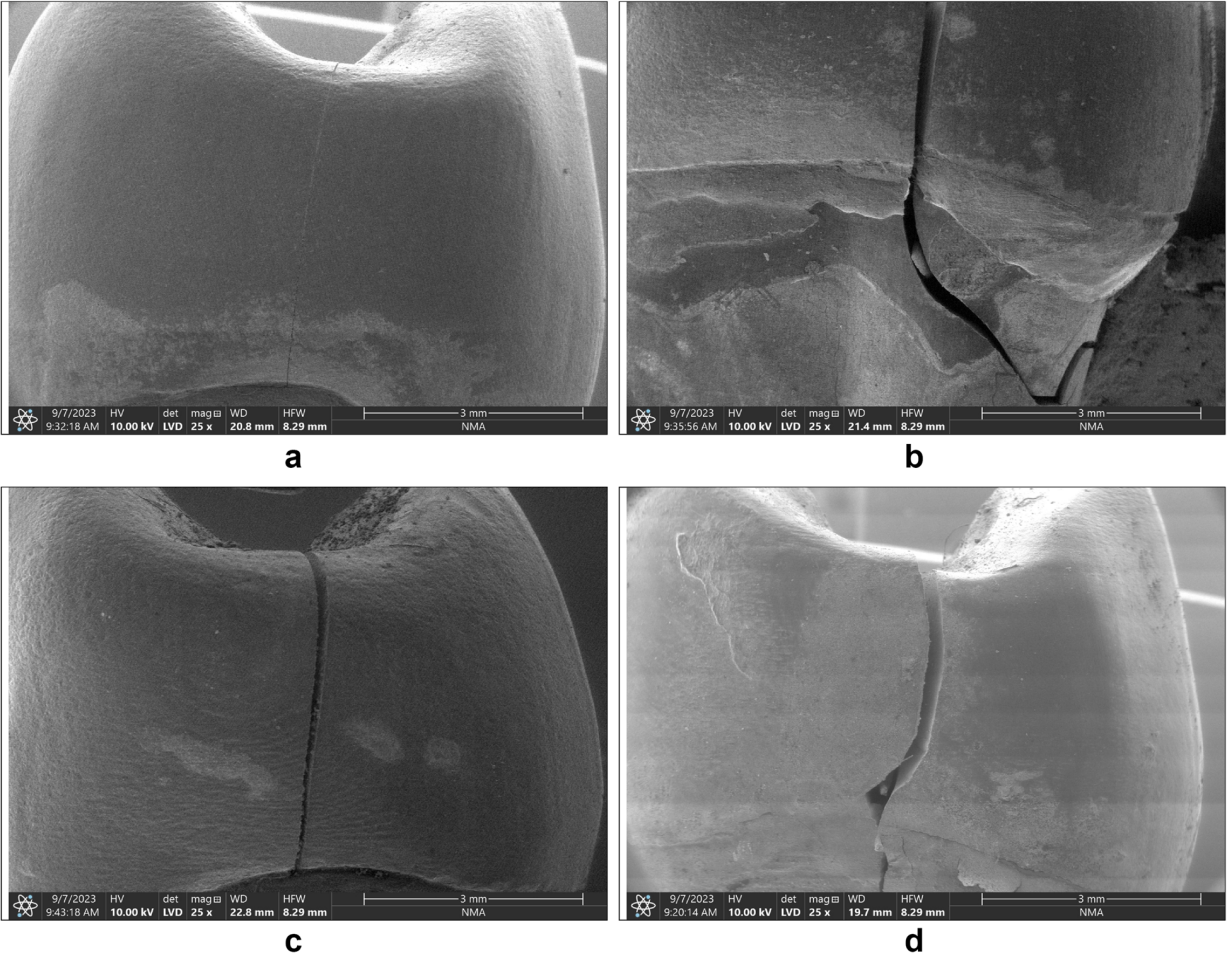
Stereomicroscope images showing different failure modes. a Cracked IPS e.max crown (Control group), b Fractured restoration in IPS e.max (Repaired group), c: Fractured restoration and tooth in IPS e.max (Repaired group), d Fractured restoration in Vita Enamic (Control group), e Fractured restoration in Vita Enamic (Repaired group), f Fractured restoration and tooth in Vita Enamic (Repaired group)

Fractured parts of each specimen were inspected using SEM and Stereomicroscope as the following:

- In control group: For IPS e.max group (LDI), 40% of specimens have cracks in the restoration, 30% of specimens suffer from a restoration fracture, and 30% of specimens have fractures in both tooth and restoration. However, in Vita Enamic control group (PICI), 20% of specimens have fractures in both tooth and restoration, 40% of specimens recorded a restoration fracture, and 40% of specimens suffer from chipped restoration failure mode.
- In repaired group: For IPS e.max group (LDR), 60% of specimens have fractures in both tooth and restoration, 10% of specimens suffer from cracks in the restoration, and 30% of specimens recorded a restoration fracture. However, in Vita Enamic repaired group (PICR), 60% of specimens have fractures in both tooth and restoration, 30% of specimens recorded a restoration fracture, and 10% of specimens suffer from chipped restoration failure mode. Based on the Chi-square test, the Chi- square value with Yates’ correction was 11.66 and this value was not statistically significant (*P*-value = 0.232).

## Discussion

Dental ceramics are considered brittle materials therefore any mechanical preparation could cause defect, crack induction, or even fracture [27]. The primary objective of the present investigation was to examine the effect of simulated root canal access on the fracture resistance of lithium disilicate, and polymer infiltrated ceramic CAD-CAM single restorations.

In the current investigation, natural teeth were utilized instead of typodont teeth or acrylic resin dies because of their strength, modulus of elasticity, and bonding properties, which better resemble the clinical setting. For standardization of crown preparation computer numerical control (CNC) machine was utilized to make preparation for all teeth observing the scientifically recommended standards for ceramic crown preparation including a finish line with a 1.0 mm circumferential chamfer, 1.5 mm axial preparation, 12° occlusal convergence angle, and 2.0 mm occlusal preparation [21].

In the current study lithium disilicate (LD) glass ceramics and polymer infiltrated ceramics (PIC) were chosen as the tested materials. According to extensive vivo and vitro investigations, lithium disilicate glass ceramic was utilized for several years as a clinically effective adhesive restoration. These investigations also support the utilization of lithium disilicate glass ceramic as a benchmark by which other recently launched CAD-CAM hybrid ceramics compared to it [28–31]. Using dual-network architecture that blends the most desirable aspects of both ceramic and resin to provide an incorporated crack stop activity, Vita Enamic possesses an elasticity that is similar to dentin and strong bonding properties. The primary ceramic structure is strengthened by a polymer network [32].

A conservative oval shaped access cavity preparation was controlled in all restorations by using a custom-made template [21] combined with metal inlay with a 5.0 mm depth, that was deemed sufficient for making access through the cemented ceramic crowns.

The absence of a PDL simulation may lead to differences in fracture behavior compared to clinical situations. Specifically, teeth embedded solely in epoxy resin may exhibit different fracture patterns than those embedded in a simulated periodontium. This is particularly relevant when considering catastrophic fractures, as the mechanical properties of the PDL would likely absorb some forces that could otherwise lead to fractures in a rigid medium like epoxy resin. Silicone materials like polyether and polysiloxane have been used to substitute for PDL during laboratory simulations. However, the differences in mechanical and physical properties, layer thicknesses, and the techniques involved in the production of the artificial PDL in vitro have been found to affect the accuracy and reproducibility of the simulated PDL layer [33]. While previous studies have shown varying approaches to simulating the PDL, the decision to omit this simulation in the present study was based on a comprehensive review of the literature. Notably, Naumann et al. [34] after reviewing the designs of 69 in vitro studies investigated the fracture resistance of post-endodontic restorations, they found that a significant proportion of studies (50% of fatigue testing studies and 72% of single load fracture tests) did not incorporate a PDL layer, suggesting that this omission is not uncommon in in vitro research. Another review [25] focused on the fatigue testing parameters applied in testing lithium disilicate restoration found that only 7 studies (out of 19 studies) applied PDL simulation. Furthermore, Nawafleh et al. [35] concluded that simulating the PDL using resilient materials does not significantly affect the in vitro survival and fracture resistance of tested crowns. This supports that the absence of a PDL simulation may not critically undermine the validity of the current findings.

However, by absence of periodontal ligament simulation, the fracture behavior of teeth does not accurately replicate clinical conditions. The use of a solid embedding material alters fracture patterns, potentially influencing the frequency of class V fractures. Embedding a tooth in epoxy resin without a periodontal ligament effectively splints the tooth root, providing artificial stabilization that deviates from the natural clinical scenario.

In the present research, artificial aging by a chewing simulator was utilized by merging mechanical loads and thermal cycling to enhance the study's clinical relevance through using 98 N of a chewing force, 2.4 Nm torque, and 1.6 Hz cycle frequency. Cold/hot bath temperature: 5/55 °C with 60 sec. dwell time. The test repeated to all specimens for 118,000 cycles to mimic one year of clinical practice [25]. According to Oguz et al. [21], using 49 N of chewing force, all specimens were put through 1.2 million cycles of thermo-mechanical loading (TML) in a dual axis chewing simulator to mimic five years of clinical practice. According to Gerogianni et al. [10], all specimens were cyclically loaded for 200,000 cycles at 8 Hz in a 37 °C, moist setting. The loads ranged from 80 to 280 N, which is comparable to one year of clinical practice.

In the present research, the highest mean fracture resistance was noted for PICI (1308.71 ± 244.15 N), followed by LDI (1154.38 ± 133.83 N), then PICR (727.8 ± 240.52 N), and the lowest value was recorded with LDR (707.03 ± 298.28 N). Statistical analysis displayed that there were significant differences amongst intact and repaired restorations while no significant difference between either LD and PIC before or after repair. Those values exceed average peak masticatory force [36] wherein the occlusal force throughout clenching may reach 520–800 N (the mean 660 N) and the mean chewing force for the upper premolar region is 222–445 N (the mean 322.5 N), suggesting both materials provide restorations that withstand the highest mastication forces.

The maximum average load value utilizing Vita Enamic, in contrast to IPS e.max, could be accounted for by the polymer matrix of Vita Enamic, adhesive system, resin cement, and composite resin repair material exhibiting synergistic activity. The strong compositional similarity of this combination provides a superior bonding ability to the underneath structure, which reinforces the restoration-tooth complex and ultimately resulted in an improved fracture resistance value [37]. Because PICs have a higher amount of polymers than ceramics, they have a less elastic modulus (about the same as dentin), which allows for the absorption of functional loads via deformation. They also have higher flexural strength, fatigue resistance, and strain at failure [19, 38]. While Kelly et al. [39] showed that Vita Enamic restorations performed similarly to lithium disilicate restorations.

The result of the current study agreed with Zhang et al. [40], they studied glass and hybrid ceramic crowns and discovered that, regardless of simulated access cavity preparation, there was no significant variation in the two types of crowns'fracture resistance.

The outcomes of the present research differ from those stated by Oguz et al. [21], who found that the fracture strength of the LD crowns was greater than that of the PIC crowns. They explained this might be because of the amount of interconnecting needle-form crystals implanted within the glass matrix of lithium disilicate [41].

The reduced fracture resistance values of tested crowns after simulated access cavity preparation and thermomechanical cycling could be attributed to the process of access cavity preparation that may trigger cracks which subsequently propagated during exposure to mechanical load and fluctuation of temperature during the aging process. In addition, the composite repair material with a different modulus of elasticity than IPS e.max and Vita Enamic crowns might change stress distribution with subsequent stress concentration at weak restoration-repair interface [42].

The limitation of the present research was that the fracture stress applied during the assessment was solely vertical, which may not fully capture the complex loading scenarios, particularly oblique forces affecting the cervical region of the tooth in clinical conditions. In addition, instead of natural teeth, a stainless-steel cylinder was used as an antagonist in the thermo-mechanical aging simulation. Despite the inherent challenges in standardizing and controlling experimental variables, future research should incorporate periodontal ligament (PDL) simulation to better reflect the complex oral environment and to evaluate its impact on the mechanical properties of various ceramic restorations.

## Conclusion

Based on the findings of the present research, the subsequent conclusions could be drawn:

1. Although endodontic access cavity preparation through lithium disilicate or polymer-infiltrated ceramics negatively affects the fracture resistance of crowns, these materials have shown the ability to create long-lasting restorations that can endure masticatory forces, even after such modifications.
2. Catastrophic tooth fractures are more likely to occur following endodontic access opening for retreatment.

## Data Availability

The dataset used and analyzed data can be available from the corresponding author upon reasonable request.

## Abbreviations

LD: Lithium-disilicate
PIC: Polymer-infiltrated ceramic
LDI: Intact lithium-disilicate
PICI: Intact polymer-infiltrated ceramic
LDR: Repaired lithium-disilicate
PICR: Repaired polymer-infiltrated ceramic
FDPs: Fixed Dental Prostheses
CNC: Computer numerical control
SEM: Scanning electron microscope
